# Rhabdomyolysis caused by interaction between rosuvastatin and vadadustat: a case report

**DOI:** 10.1186/s40780-023-00281-2

**Published:** 2023-04-10

**Authors:** Keiki Sakurama, Yuki Iguchi, Sara Haruki, Yusuke Hata, Madoka Hiraga, Shinya Yumoto, Yutaka Kai

**Affiliations:** 1 Department of Pharmacy, Aso Medical Center, 1266 Kurokawa, Aso-Shi, Kumamoto-ken 869-2225 Japan; 2 Department of Internal Medicine, Aso Medical Center, 1266 Kurokawa, Aso-Shi, Kumamoto-ken 869-2225 Japan; 3 Department of Nephrology, Kumamoto University, 1-1-1, Honjo, Kumamoto Shi Chuo Ku, Kumamoto Ken 860-0811 Japan; 4 Department of Neurosurgery, Aso Medical Center, 1266 Kurokawa, Aso-Shi, Kumamoto-ken 869-2225 Japan

**Keywords:** Rhabdomyolysis, Rosuvastatin, Vadadustat, Drug interaction

## Abstract

**Background:**

Rhabdomyolysis is a potentially life-threatening disease caused by melting or necrosis of skeletal muscle cells and leakage of muscle components into the bloodstream. It has been reported that the interaction of the HMG-CoA reductase inhibitor rosuvastatin with the renal anemia drug vadadustat increases the blood concentration of rosuvastatin in vitro. In this study, we report a case of suspected rhabdomyolysis caused by the drug interaction of rosuvastatin and vadadustat in clinical practice.

**Case presentation:**

A 62-year-old male with medical records of hypertension, myocardial infarction, chronic renal failure, renal anemia, dyslipidemia, and alcoholic liver disease. The patient had been diagnosed with chronic kidney disease (CKD) at the Department of Nephrology, and treated by outpatient care with renal support therapy for the past two years. On X-63 day, his prescription was rosuvastatin (10 mg/day) and a continuous erythrocyte-stimulating agent, epoetin beta pegol (genetical recombination, 100 μg). X-Day 0, blood tests revealed creatine phosphokinase (CPK) 298 U/L, serum creatinine (SCr) 5.26 mg/dL, and hemoglobin (Hb) 9.5 g/dL; thus, the prescription was changed from epoetin beta pegol 100 μg to vadadustat 300 mg/day. On X + day 80, a prescription for a diuretic (azosemide 15 mg/day) was added for swelling of the lower extremities. On X + day 105, we found CPK 16,509 U/L, SCr 6.51 mg/dL, and Hb 9.5 g/dL. The patient was diagnosed as rhabdomyolysis and hospitalized. After hospitalization, rosuvastatin and vadadustat were discontinued and we administered intravenous fluids. Thereafter, CPK and SCr values of the patient improved. On X + day 122, CPK improved to 29 U/L, SCr to 2.6 mg/dL, and Hb to 9.6 g/dL, and he was discharged on X + day 124. At discharge, rosuvastatin 2.5 mg/day was resumed. A blood test on X + day 133 showed CPK 144 U/L and SCr 4.2 mg/dL.

**Conclusion:**

We experienced a case of rhabdomyolysis caused by drug interactions between rosuvastatin and vadadustat.

## Introduction

Rhabdomyolysis is a potentially fatal disease caused by melting or necrosis of skeletal muscle cells and the leakage of muscle components into the bloodstream.

Previous studies reported that the drug interaction of rosuvastatin [1], an HMG-CoA reductase inhibitor, and vadadustat [2, 3], a therapeutic drug for Hypoxia Inducible Factor Prolyl Hydroxylase renal anemia, increases the blood concentration of rosuvastatin in vitro [4, 5]. In this study, we report a case of rhabdomyolysis [6] caused by the drug-interaction of rosuvastatin and vadadastat in clinical practice.

## Case presentation

A 62-year-old male with medical records of hypertension, myocardial infarction, chronic renal failure, renal anemia, dyslipidemia, and alcoholic liver disease. The patient had been diagnosed with CKD at the Department of Nephrology, and treated by outpatient care with renal support therapy for the past two years. On X-63 day, his prescription was rosuvastatin (10 mg/day) and a continuous erythrocyte-stimulating agent, epoetin beta pegol [7] (genetical recombination, 100 μg). Blood tests revealed SCr 3.75 mg/dL, Hb 9.5 g/dL, high density lipoprotein cholesterol (HDL-c) 69 mg/dL, and low density lipoprotein cholesterol (LDL-c) 21 mg/dL. X-day 0, blood tests revealed CPK 298 U/L, SCr 5.26 mg/dL, Hb 9.5 g/dL, HDL-c 62 mg/dL, and LDL-c 16 mg/dL, so the prescription was changed from epoetin beta pegol 100 μg to vadadustat 300 mg/day. On X + day 80, a prescription for a diuretic (azosemide [8] 15 mg/day) was added for swelling of the lower extremities. Muscle pain in both legs appeared on around X + 80 day. Furthermore, at the time of admission, the patient had persistent muscle pain in both legs, further muscle weakness, and increase of CPK level. Reddish-brown urine, possibly myoglobinuria, was also observed. Therefor the patient was diagnosis as rhabdomyolysis.

On X + day 105 at his regular clinic visit, blood tests showed CPK 16,509 U/L, SCr 6.51 mg/dL, Hb 9.5 g/dL, HDL-c 33 mg/dL, and LDL-c 16 mg/dL. The patient was diagnosed with rhabdomyolysis and hospitalized. On admission, the pharmacist suggested to the doctor that the discontinuation of vadadustat and rosuvastatin to the physician as a possible cause of the elevated CPK levels due to drug interactions. As a result, both drugs were discontinued and the patient was administered intravenous fluids. Thereafter, CPK and SCr values improved. The pharmacist suggested to the doctor epoetin beta pegol, which had been previously administered for renal anemia, since the CPK level had decreased. Furthermore, the doctor asked the pharmacist to resume rosuvastatin because of ischemic heart disease. The pharmacist informed the doctor that statins alone have few side effects such as rhabdomyolysis [9], and the drug was resumed in small doses in consideration of safety.

**Fig. 1:**
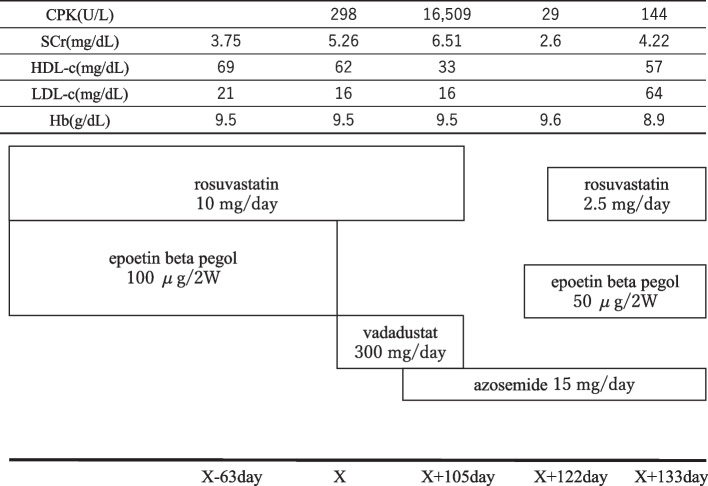
Clinical course

On X + day 122, CPK improved to 29 U/L, SCr 2.6 mg/dL, and Hb 9.6 g/dL, and he was discharged on X + day 124. At discharge, rosuvastatin 2.5 mg/day was resumed. A blood test on X + day 133 showed CPK 144 U/L SCr 4.22 mg/dL, Hb 8.9 g/dL, HDL-c 57 mg/dL, and LDL-c 64 mg/dL (Fig. 1).

## Discussion and conclusion

Rhabdomyolysis is a potentially life-threatening condition caused by the melting and necrosis of skeletal muscle cells and the leakage of muscle components into the blood [6].

The diagnostic criteria for rhabdomyolysis refer to a CPK level of at least five times the reference value, and symptoms include myalgia and myoglobinuria. Treatment includes discontinuation of the causative drugs, if any, and application of infusion fluids [10]. Congenital rhabdomyolysis and acquired rhabdomyolysis are known to be causes of rhabdomyolysis. Acquired rhabdomyolysis has been reported to be caused by excessive exercise [11], alcohol [12], liver and kidney damage [13, 14] and especially HMG-CoA reductase inhibition (statins) [15]. Statin-induced rhabdomyolysis has been reported to occur in interactions with drugs such as fibrates and cyclosporine [16], although few cases have been reported alone. In addition, rhabdomyolysis has been reported to have occurred because of increased blood levels of statins due to drug interactions [17]. It has been reported that in vitro a combination of rosuvastatin and vadadustat increased the plasma concentration of rosuvastatin [4, 5] a substrate of breast cancer resistance protein (BCRP) [18], due to the inhibitory effect of vadadustat on BCRP.

In this study, we experienced a case of rhabdomyolysis that was thought to be caused by the interaction between the statin rosuvastatin and the renal anemia drug vadadustat. Since statins have evidence for secondary prevention of ischemic heart disease, rosuvastatin was taken by this patient aiming for LDL-c < 70 mg/dL [19]. And it was thought that the plasma concentration of rosuvastatin was increased by vadadustat, which led to an increase in CKP and rhabdomyolysis. Furthermore, hepatic and renal impairment, alcohol intake, excessive exercise, and dehydration due to diuretics may have contributed to rhabdomyolysis. In addition, after resuming only rosuvastatin at the time of discharge, CPK and SCr did not increase, suggesting that rhabdomyolysis was caused by the combination of rosuvastatin and vadadustat. Judging from this case, it would be advisable to administer rosuvastatin carefully when it is combined with vadadustat, taking into account drug interactions and patient background.

Vadadustat taken alone has been reported to have no effect on cholesterol levels [20]. When vadadustat and rosuvastatin were co-administered, LDL-c did not change, but HDL-c decreased. Therefore, the combination of rosuvastatin and vadadustat may influence cholesterol levels. Though the cause was not identified in this study. Further verification is necessary in the future.

In conclusion, the pharmacist was aware from the package insert of vadadustat and rosuvastatin as stating “concomitant use is cautioned because the Cmax and AUC of rosuvastatin may increase with concomitant use” [4], in other words, it causes the drug interaction between them resulting in an increase in the Cmax and AUC of rosuvastatin. This predicted an increase in CPK and may have caused rhabdomyolysis from persistent myalgia and myoglobinuria. This was indicated to the doctor.

## Data Availability

The datasets used and/or analyzed during the current study are available from the corresponding author upon reasonable request.
