# Correction to: Rhabdomyolysis caused by interaction between rosuvastatin and vadadustat: a case report

**DOI:** 10.1186/s40780-023-00285-y

**Published:** 2023-05-05

**Authors:** Keiki Sakurama, Yuki Iguchi, Sara Haruki, Yusuke Hata, Madoka Hiraga, Shinya Yumoto, Yutaka Kai

**Affiliations:** 1 Department of Pharmacy, Aso Medical Center, 1266 Kurokawa, Aso-Shi, Kumamoto-ken, 869-2225 Japan; 2 Department of Internal Medicine, Aso Medical Center, 1266 Kurokawa, Aso-Shi, Kumamoto-ken, 869-2225 Japan; 3 Department of Nephrology, Kumamoto University, 1-1-1, Honjo, Kumamoto Shi Chuo Ku, Kumamoto-ken, 860-0811 Japan; 4 Department of Neurosurgery, Aso Medical Center, 1266 Kurokawa, Aso-Shi, Kumamoto-ken, 869-2225 Japan

Journal of Pharmaceutical Health Care and Sciences (2023) 9:13

10.1186/s40780-023-00281-2

The original publication of this article contained an incorrect title.

The correct and incorrect title are listed below, the original article has been updated.

## Incorrect

Rhabdomyolysis caused by interaction between rosuvastatin and vadadustat: a case control study

## Correct

Rhabdomyolysis caused by interaction between rosuvastatin and vadadustat: a case report

